# Plasma Metabolites Associated with Coffee Consumption: A Metabolomic Approach within the PREDIMED Study

**DOI:** 10.3390/nu11051032

**Published:** 2019-05-08

**Authors:** Christopher Papandreou, Pablo Hernández-Alonso, Mònica Bulló, Miguel Ruiz-Canela, Edward Yu, Marta Guasch-Ferré, Estefanía Toledo, Courtney Dennis, Amy Deik, Clary Clish, Cristina Razquin, Dolores Corella, Ramon Estruch, Emilio Ros, Montserrat Fitó, Fernando Arós, Miquel Fiol, José Lapetra, Cristina Ruano, Liming Liang, Miguel A. Martínez-González, Frank B. Hu, Jordi Salas-Salvadó

**Affiliations:** 1 Human Nutrition Unit, Faculty of Medicine and Health Sciences, Institut d’Investigació Sanitària Pere Virgili, Rovira i Virgili University, 43201 Reus, Spain; papchris10@gmail.com (C.P.); pablo1280@gmail.com (P.H.-A.); mguasch@hsph.harvard.edu (M.G.-F.); 2 CIBER Fisiopatología de la Obesidad y Nutrición (CIBEROBN), Instituto de Salud Carlos III, 28029 Madrid, Spain; mcanela@unav.es (M.R.-C.); etoledo@unav.es (E.T.); crazquin@unav.es (C.R.); dolores.corella@uv.es (D.C.); RESTRUCH@clinic.ub.es (R.E.); eros@clinic.ub.es (E.R.); MFito@imim.es (M.F.); lfaborau@gmail.com (F.A.); miquelfiol@yahoo.es (M.F.); jlapetra@ono.com (J.L.); cristina.ruano@ulpgc.es (C.R.); lliang@hsph.harvard.edu (L.L.); mamartinez@unav.es (M.A.M.-G.); 3 University of Navarra, Department of Preventive Medicine and Public Health, IdiSNA, 31009 Pamplona, Spain; 4 Department of Nutrition, Harvard T.H. Chan School of Public Health, Boston, MA 02115, USA; edy356@mail.harvard.edu (E.Y.); nhbfh@channing.harvard.edu (F.B.H.); 5 Department of Epidemiology, Harvard T.H. Chan School of Public Health, Boston, MA 02115, USA; 6 Broad Institute of MIT and Harvard University, Cambridge, MA 02142, USA; cdennis@broadinstitute.org (C.D.); adeik@broadinstitute.org (A.D.); clary@broadinstitute.org (C.C.); 7 Department of Preventive Medicine, University of Valencia, 46010 Valencia, Spain; 8 Department of Internal Medicine, Department of Endocrinology and Nutrition Institut d’ Investigacions Biomediques August Pi Sunyer (IDIBAPS), Hospital Clinic, University of Barcelona, 08007 Barcelona, Spain; 9 Lipid Clinic, Department of Endocrinology and Nutrition Institut d’Investigacions Biomediques August Pi Sunyer (IDIBAPS), Hospital Clinic, University of Barcelona, 08007 Barcelona, Spain; 10 Cardiovascular Risk and Nutrition Research Group (CARIN), Hospital del Mar Research Institute (IMIM), 08003 Barcelona, Spain; 11 Department of Cardiology, University Hospital of Álava, 01009 Vitoria, Spain; 12 Illes Balears Health Research Institute (IdISBa), Hospital Son Espases, 07120 Palma de Mallorca, Spain; 13 Department of Family, Research Unit, Distrito Sanitario Atención Primaria Sevilla, 41013 Sevilla, Spain; 14 Department of Clinical Sciences, University of Las Palmas de Gran Canaria, 35001 Las Palmas, Spain; 15 Department of Biostatistics, Harvard T.H. Chan School of Public Health, Boston, MA 02115, USA; 16 Channing Division for Network Medicine, Department of Medicine, Brigham and Women’s Hospital and Harvard Medical School, MA 02115, USA

**Keywords:** coffee, caffeine, plasma, metabolomics, PREDIMED

## Abstract

Few studies have examined the association of a wide range of metabolites with total and subtypes of coffee consumption. The aim of this study was to investigate associations of plasma metabolites with total, caffeinated, and decaffeinated coffee consumption. We also assessed the ability of metabolites to discriminate between coffee consumption categories. This is a cross-sectional analysis of 1664 participants from the PREDIMED study. Metabolites were semiquantitatively profiled using a multiplatform approach. Consumption of total coffee, caffeinated coffee and decaffeinated coffee was assessed by using a validated food frequency questionnaire. We assessed associations between 387 metabolite levels with total, caffeinated, or decaffeinated coffee consumption (≥50 mL coffee/day) using elastic net regression analysis. Ten-fold cross-validation analyses were used to estimate the discriminative accuracy of metabolites for total and subtypes of coffee. We identified different sets of metabolites associated with total coffee, caffeinated and decaffeinated coffee consumption. These metabolites consisted of lipid species (e.g., sphingomyelin, phosphatidylethanolamine, and phosphatidylcholine) or were derived from glycolysis (alpha-glycerophosphate) and polyphenol metabolism (hippurate). Other metabolites included caffeine, 5-acetylamino-6-amino-3-methyluracil, cotinine, kynurenic acid, glycocholate, lactate, and allantoin. The area under the curve (AUC) was 0.60 (95% CI 0.56–0.64), 0.78 (95% CI 0.75–0.81) and 0.52 (95% CI 0.49–0.55), in the multimetabolite model, for total, caffeinated, and decaffeinated coffee consumption, respectively. Our comprehensive metabolic analysis did not result in a new, reliable potential set of metabolites for coffee consumption.

## 1. Introduction

Coffee, a widely consumed beverage worldwide [1], has been associated with both beneficial and detrimental effects on health-related outcomes, although harmful associations have been shown to be largely nullified by adequate adjustment for smoking [2]. The most commonly consumed types of coffee, caffeinated and decaffeinated, have consistently been associated with a lower risk of type 2 diabetes (T2D) [3]. However, these findings are based on self-reported dietary assessment methods that might be subject to some degree of misclassification or measurement error [4].

Metabolomics has the potential to advance nutritional epidemiology by objectively measuring metabolic products of foods, and might therefore better accurately reflect food exposure [5]. A comprehensive metabolite profiling may also provide a deeper understanding of metabolic response to foods providing new functional insight to their role in health. Particularly, coffee contains a variety of compounds, many of which may impact metabolic pathways related to disease development or prevention.

In this regard, coffee consumption has been found to be positively associated with two classes of sphingomyelins and negatively associated with long- and medium-chain acylcarnitines in plasma among 284 men of the KORA study population [6]. In a prospective study of 1610 EPIC-Potsdam participants, coffee consumption was inversely associated with one diacylphosphatidylcholine in both sexes and phenylalanine in men, whereas in women, coffee consumption was positively associated with three acyl-alkyl-phosphatidylcholine species [7]. Other metabolites related to coffee exposure include hydroxycinnamate derivatives, phenolic acid derivatives and dimethoxycinnamic acids [8,9,10].

Recently, a 3-stage coffee trial found 115 serum metabolites significantly associated with coffee consumption among 47 participants [11]. Five metabolic pathways were significantly enriched: (a) xanthine, (b) benzoate, (c) steroid, (d) fatty acid (acyl choline), and (e) endocannabinoid [11]. The same research team extended this work to analysis of the lipidomic changes in response to coffee consumption and found that coffee may induce alterations in glycerophospholipid metabolism [12]. However, to date, limited or no metabolomic-analysis has been conducted using combinations of different metabolomic platforms to cover a wide range of metabolites and examine their association with total and subtypes of coffee consumption.

Other studies examining the ability of metabolites to discriminate between coffee consumption categories suggested that trigonelline could be a useful marker of coffee consumption [13]. Regarding caffeine, few studies have assessed its discriminating accuracy with inconsistent results [14,15]. Caffeine is also found in tea, soft drinks, and other food items [16] making it difficult to control for this.

Using a validated multiplatform metabolomics analysis, cross-sectional associations between plasma levels of identified metabolites with self-reported total, caffeinated, or decaffeinated coffee consumption were examined in participants of the PREDIMED (Prevención con DietaMediterránea) study. The ability of metabolites to discriminate between total and the aforementioned types of coffee consumption categories was also investigated.

## 2. Materials and Methods

### 2.1. Study Design

This study is a cross-sectional evaluation of baseline data from two nested case-cohort studies [17] within the PREDIMED trial (ISRCTN35739639), a primary prevention cardiovascular disease (CVD) trial conducted in 7447 participants free of cardiovascular events at baseline but at high cardiovascular risk. Participants were men (55–80 years) and women (60–80 years) without CVD at baseline and fulfilling at least one of the two following criteria; presence of T2D or three or more major cardiovascular risk factors. A detailed description of the PREDIMED trial can be found elsewhere [18,19]. In brief, 7447 participants were randomly assigned to a Mediterranean diet regime, supplemented with extra-virgin olive oil; a Mediterranean diet supplemented with mixed nuts, or a control diet consisting of advice to reduce fat intake. All participants provided written informed consent, and the study protocol and procedures were approved according to the ethical standards of the Declaration of Helsinki. The Institutional Review Board (IRB) of Hospital Clinic (Barcelona, Spain) approved the study protocol on July 2002. This IRB is accredited by the US Department of Health and Human Services (DHHS). Later, the IRBs of all other centers also approved the protocol. 

### 2.2. Subjects Selection

**Figure 1:**
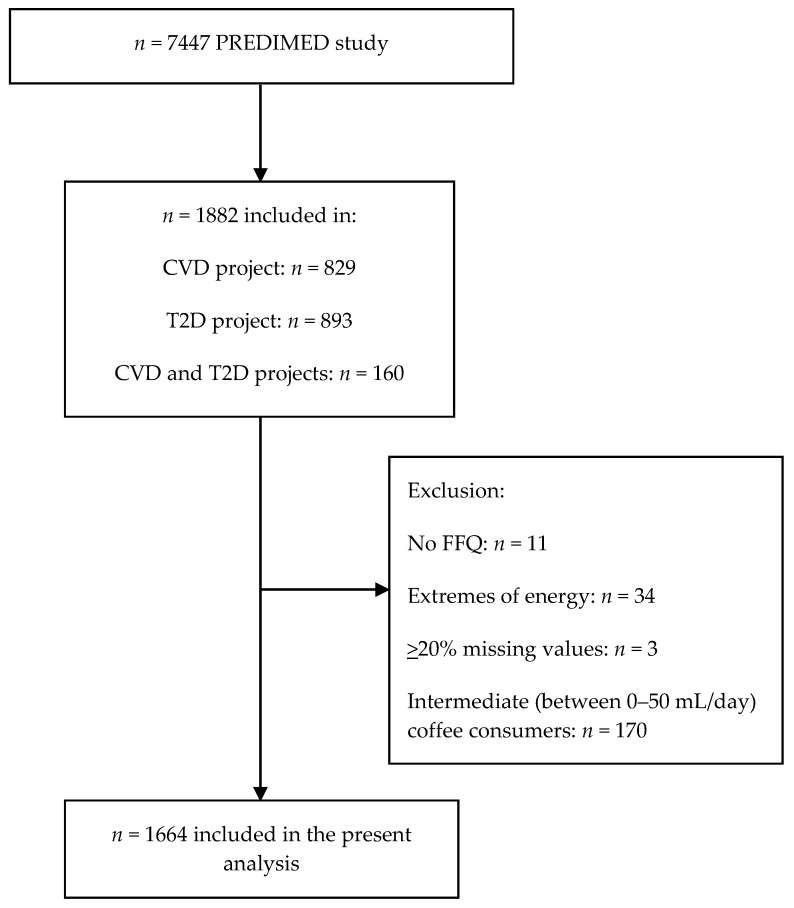
Flowchart of study participants. *, Extremes of energy are defined as out of the range 800–4000 Kcal/day in males and 500–3500 Kcal/day in females. Abbreviations: CVD, cardiovascular disease; FFQ, food frequency questionnaire; T2D, type 2 diabetes.

For the present study, 1871 out of 1882 PREDIMED subjects with available metabolomics data from the CVD [17] and T2D [20] projects, who completed a validated semiquantitative 137-item food frequency questionnaire (FFQ) were included [21]. Nutrient and energy intakes were calculated using Spanish food composition tables [22]. Participants (*n* = 34) who had extremes daily energy intake (<500 or >3500 kcal/day for women and <800 or >4000 kcal/day for men) were excluded from the present analysis as well as those (*n* = 3) with equal or more than 20% missing values in metabolites leaving 1834 subjects for further analyses (Figure 1). Two questions from the FFQ concerned the average consumption of coffee with and without caffeine during the previous 12 months. Validity of the FFQ for coffee, estimated by the intraclass correlation coefficient was 0.75. When coffee was treated as categorical variable (consumers vs. nonconsumers) the Kappa coefficient was 0.58. Ten categories were provided, ranging from “never or almost never” to “6 times/day or more”. Furthermore, the usual portion size was assessed, with 1 cup defined as 50 mL. For the present analyses, the total coffee consumption was calculated as the sum of caffeinated and decaffeinated coffee. Due to the characteristics of the population being at high risk of CVD and >86% of them reported to have hypertension, the majority of them (*n* = 363) presented daily coffee consumption of 50 mL per day followed by 241 participants consuming 125 mL per day. Subsequently, coffee consumers were selected with at least 50 mL per day coffee consumption. We also separated caffeinated and decaffeinated coffee consumers and excluded overlapping cases in order to avoid their confounding effect on associations between metabolites and types of coffee consumption. There were 512 caffeinated coffee consumers and 721 decaffeinated coffee consumers. We categorized participants into (1) two groups of total coffee, 285 nonconsumers (0 mL coffee per day) vs. 1379 consumers (50 mL or more coffee per day); (2) two groups of caffeinated coffee, 285 nonconsumers (0 mL coffee per day) vs. 512 consumers (50 mL or more coffee per day); and (3) two groups of decaffeinated coffee, 285 nonconsumers (0 mL coffee per day) vs. 721 consumers (50 mL or more coffee per day). Finally, the consumption of other sources of caffeine, including tea and cocoa was relatively low (*n* = 284 and *n* = 115, respectively) among the 1664 study participants.

### 2.3. Metabolomics

Fasting (for ≥8 h) plasma EDTA samples were collected from subjects and stored at −80 °C. Pairs of samples for each participant were randomly ordered and analyzed using two liquid chromatography tandem mass spectrometry (LC–MS) methods to measure polar metabolites and lipids as described previously [23,24,25]. Briefly, amino acids and other polar metabolites were profiled with a Shimadzu Nexera X2 U-HPLC (Shimadzu Corp.) coupled to a Q Exactive mass spectrometer (ThermoFisher Scientific). Metabolites were extracted from plasma (10 μL) using 90 μL of 74.9:24.9:0.2 (*v*/*v*/*v*) of acetonitrile/methanol/formic acid containing stable isotope-labeled internal standards (valine-d8 (Sigma-Aldrich) and phenylalanine-d8 (Cambridge Isotope Laboratories)). The samples were centrifuged (10 min; 9000× *g*; 4 °C) and the supernatants were injected directly on to a 150 × 2-mm, 3-μm Atlantis HILIC column (Waters). The column was eluted isocratically at a flow rate of 250 μL/min with 5% mobile phase A (10 mmol ammonium formate/L and 0.1% formic acid in water) for 0.5 min followed by a linear gradient to 40% mobile phase B (acetonitrile with 0.1% formic acid) over 10 min. MS analyses were carried out using electrospray ionization in the positive-ion and full-scan spectra were acquired over 70–800 m/z. Lipids were profiled using a Shimadzu Nexera X2 U-HPLC (Shimadzu Corp.; Marlborough, MA) coupled to an Exactive Plus orbitrap mass spectrometer (Thermo Fisher Scientific; Waltham, MA). Lipids were extracted from plasma (10 μL) using 190 μL of isopropanol containing 1,2-didodecanoyl-sn-glycero-3-phosphocholine (Avanti Polar Lipids; Alabaster, AL) as an internal standard. Lipid extracts (2 μL) were injected onto a 100 × 2.1 mm, 1.7 μm ACQUITY BEH C8 column (Waters; Milford, MA). The column was eluted isocratically with 80% mobile phase A (95:5:0.1 *v*/*v*/*v* 10 mM ammonium acetate/methanol/formic acid) for 1 min followed by a linear gradient to 80% mobile-phase B (99.9:0.1 *v*/*v* methanol/formic acid) over 2 min, a linear gradient to 100% mobile phase B over 7 min, then 3 min at 100% mobile-phase B. MS analyses were carried out using electrospray ionization in the positive ion mode using full scan analysis over 200 to 1100 m/z. Raw data were processed using Trace Finder version 3.1 and 3.3 (Thermo Fisher Scientific) and Progenesis QI (Nonlinear Dynamics; Newcastle upon Tyne, UK). Polar metabolite identities were confirmed using authentic reference standards and lipids were identified by head group and total acyl carbon number and total acyl double bond content. To enable assessment of data quality and to facilitate data standardization across the analytical queue and sample batches, pairs of pooled plasma reference samples were analyzed at intervals of 20 study samples. One sample from each pair of pooled references served as a passive QC sample to evaluate the analytical reproducibility for measurement of each metabolite while the other pooled sample was used to standardize at using a “nearest neighbor” approach. Standardized values were calculated using the ratio of the value in each sample over the nearest pooled plasma reference multiplied by the median value measured across the pooled references. Plasma levels of 398 metabolites were measured.

### 2.4. Statistical Analysis

Baseline characteristics of study participants were described according to nonconsumers and frequent total coffee consumers as means (SD) for quantitative traits and percentages for categorical variables. From the 398 metabolites measured in the present study, 11 metabolites were removed due to high number of missing values (i.e., ≥20%), leaving 387 metabolites for further analysis. Missing values of individual metabolites were imputed (in those metabolites with less than 20% of missing values) using the random forest imputation approach (“missForest” R package). Missing values are those determinations that were below the limit of detection. The levels of metabolites were normalized and scaled to multiples of 1 SD with the rank-based inverse normal transformation. Due to the high dimensionality and collinear nature of the data, logistic regression with elastic net penalty was implemented in the “glmnet” R package (alpha = 0.5) to build a discrimination model for frequent coffee consumption. We performed 10-fold cross-validation to find the optimal value of the tuning parameter that result in a mean squared error within 1-SD of the minimum [26]. The discrimination accuracy was examined based on parameters of lamda.min. The discrimination model scores were computed as the weighted sum of all metabolites with weights equal to the regression coefficients from the discrimination models. To estimate the discrimination accuracy we split the data into 90% set and 10% set. Within the 90% set, we used the same elastic net procedure we used to build the model. Another 10-fold cross-validation was used to tune the model parameters. Then, we used the outer 10% set to evaluate the model built at the previous step. This procedure guarantees that the outer 10% set is completely separated from the model building procedure, thus the discriminative accuracy estimated in this step is unbiased. We then repeated all these steps for 10 times and averaged their discriminative accuracy in the 10% set. Since each of them is unbiased estimate of discriminative accuracy, the average is also unbiased. Logistic regression analysis was also performed and the derived coefficients were used to build models consisting of either only caffeine or 5-acetylamino-6-amino-3-methyluracil as concerns total and caffeinated coffee. The area under curve (AUC) was used to assess the discriminating power of the discrimination models for total and subtypes of coffee consumption. We compared the AUCs of the models including metabolites selected from elastic net regression analysis with a model including only caffeine or 5-acetylamino-6-amino-3-methyluracil using a nonparametric method. All analyses were performed using R statistical package 3.4.3 (www.r-project.org) (R Development Core Team, 2012).

## 3. Results

### 3.1. Participants’ Characteristics

Participants’ characteristics are summarized in Table 1. The mean age of participants at baseline was 67.1 years and the mean BMI was 29.9 kg/m^2^. As compared with nonconsumers, those participants consuming coffee were more likely to be men, current smokers, and to have a higher prevalence of hypercholesterolemia in addition to higher BMI (Table 1).

### 3.2. Associations between Plasma Metabolites and Total Coffee Consumption

Table 2 and Table 3 show selected metabolites (*n* = 11) ranked from the highest to the lowest elastic net positive and negative regression coefficients for total coffee consumption. Five-acetylamino-6-amino-3-methyluracil (AAMU), caffeine, cotinine, and sphingomyelin 24:0 were positively associated, while proline betaine, kynurenic acid, glycocholate, lactate, glyco-deoxy-chenodeox, sucrose, and 7-methylguanine were negatively associated with total coffee consumption.

Metabolites (*n* = 10) ranked from the highest to the lowest elastic net positive and negative regression coefficients for caffeinated coffee consumption are displayed in Table 2 and Table 3, respectively. Positive regression coefficients were found for four metabolites, caffeine, AAMU, sphingomyelin 24:0 and cotinine; while negative for sucrose, proline betaine, acetaminophen, one lyso-phosphatidylethanolamine (LPE) (16:0), piperine, and hypoxanthine.

Regarding decaffeinated coffee consumption, positive regression coefficients were found for five metabolites, including hydroxyhippurate, alpha-glycerophosphate, one sphingomyelin (24:0), hippurate, and phosphatidylcholine 40:6, while negative for LPE 16:0, phosphocreatine, and allantoin.

### 3.3. Discrimination of Total and Types of Coffee Consumption

**Figure 2:**
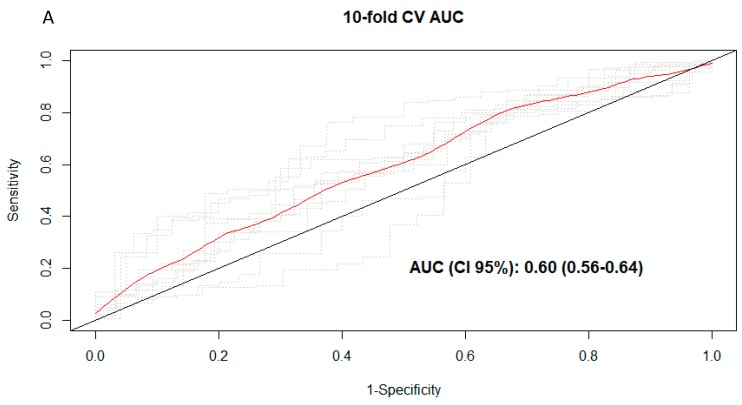

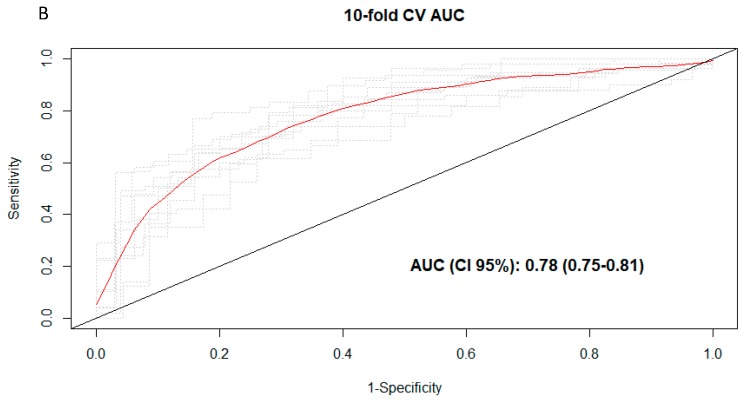
(**A**) Cross-validated receiver operating characteristic (ROC) curves for total coffee consumption. (**B**) en-fold cross-validated ROC curves for caffeinated coffee consumption. Red curve represent the 10-fold CV ROC curve, whereas dotted lines show ROC curves for each of the 10 iterations using the training-validation (90–10%) pair datasets.

To explore the discriminative ability of the multimetabolite models, AUC analyses were carried out. The AUCs were 0.60 (95% CI 0.56–0.64) (Figure 2A), 0.78 (95% CI 0.75–0.81) (Figure 2B), and 0.52 (95% CI 0.49–0.55) in the multimetabolite models for total, caffeinated, and decaffeinated coffee consumption, respectively. The AUC was significantly higher for total coffee (AUC: 0.66 (95% CI 0.62–0.71)) when only caffeine was kept in the model, *p* = 0.014. Significantly higher AUC was also found as compared to multimetabolite score for caffeinated coffee consumption (AUC: 0.79 (95% CI 0.77–0.82)), *p* = 0.029. On the other hand, AAMU had significantly lower AUCs for total (0.56 (95% CI 0.53–0.60)) and caffeinated (0.66 (95% CI 0.60–0.73)) coffee.

## 4. Discussion

In the present study, specific metabolites associated with total, caffeinated and decaffeinated coffee consumption were identified among participants of the PREDIMED study. It was also demonstrated that caffeine alone had better discriminative ability for total and caffeinated coffee consumption as compared to a set of metabolites which also included caffeine, and therefore may be used as biomarker of consumption complementing traditional dietary assessment tools. Similarly, a previous cross-sectional study found that urine caffeine is useful (AUC: 0.85) to distinguish between those subjects drinking less than one caffeinated coffee per week versus at least one caffeinated coffee [14]. However, in the aforementioned study risk of overfitting is high due to the lack of internal or external validation [15].On the other hand, a previous metabolomics study showed that caffeine was much less performant to predict coffee consumption in a cohort study than other coffee metabolites alone or in combination [27]. We found a low discrimination of decaffeinated coffee categories from the multimetabolite model. Recently, a cross-sectional study investigating how accurately could plasma trigonelline discriminate consumers from nonconsumers of coffee suggested that this plant alkaloid may serve as a useful marker of coffee consumption (AUC:0.92) [13]. However, the lack of internal or external validation limits the generalizability of this marker.

A consistent set of three metabolites was positively associated with total and caffeinated coffee consumption. Plasma caffeine and one of its major metabolites, AAMU, reflect caffeine exposure [28]. Cotinine, the main metabolite of nicotine [29] could confirm the higher prevalence of tobacco exposure in frequent coffee consumers in our study. Higher cigarette consumption has been suggested to causally increase coffee consumption [30].

One sphingomyelin (SM 24:0) was also positively associated with total, caffeinated and decaffeinated coffee consumption. A previous study among German elders found two classes of SMs, one containing a hydroxygroup and the other having an additional carboxygroup to be directly associated with coffee consumption [6]. Furthermore, we found one phosphatidylcholine (C40:6) positively associated with decaffeinated coffee consumption. A cross-sectional analysis in participants in the EPIC-Potsdam study suggests associations of coffee consumption with certain acyl-alkyl-phosphatidylcholines, including C40:6 [7].

The positive association found in the present study between alpha-glycerophosphate (alpha-GP) and decaffeinated coffee is interesting since this metabolite is formed from glycolysis and previous experiments using perfused rat adipocytes propose a model, where alpha-GP combines with long-chain coenzyme A (LC-CoA) to form triacylglycerol (TAG), thus decreasing LC-CoA and its inhibition of TAG lipases leading to release of free fatty acids and glycerol [31]. Finally, hippurate, a gut microbial metabolite of polyphenol metabolism, has been previously associated with the consumption of polyphenol-rich foods including coffee [32].

Concerning metabolites negatively associated with total coffee consumption, proline betaine had the highest coefficient in our study. This metabolite is a biomarker for citrus fruit consumption [33]. Caffeine has been considered as a diuretic, mainly because of its caffeine content [34], and this action could increase the renal excretion of proline betaine as well as ofpiperine, the major bioactive component of pepper [35], acetaminophen also known as paracetamol and 7-methylguanine. The latter metabolite is considered to be a useful marker of DNA damage caused by nitrosamines in tobacco smoke and has been found significantly higher in the urine of smokers than in non-smokers [36]. Sucrose was also recorded to be negatively associated with total and caffeinated coffee consumption and its levels in blood may reflect gastric permeability to sucrose [37].

Inverse associations between coffee consumption and systemic levels of a number of different inflammatory markers including interferon-γ (IFN-γ) have been reported [38] and IFN-γ-mediated breakdown of tryptophan to kynurerine and downstream kynurenine metabolites like kynurenic acid could be counteracted by the consumption of coffee [39]. This could partially explain the inverse association between kynurenic acid and coffee consumption in our study.

Plasma glycocholate levels were also recorded to be negatively associated with total coffee drinking in our study. Glycocholic acid is a conjugated bile acid (cholic acid with glycine) synthesized in the liver and is involved in dietary lipids emulsification and cholesterol absorption [40]. Whether coffee consumption has an effect on glycocholic acid synthesis and/or release into circulation is unknown and needs further investigation. A disruption in bile acid synthesis could lead to cholesterol homeostasis dysregulation [41]. Meta-analyses of clinical trials of the effect of coffee consumption on blood lipids suggest increases in cholesterol levels [42,43], which is in line with our findings.

The inverse association between coffee consumption and plasma lactate levels merits discussion. Caffeine is known to have a stimulating effect on the central nervous system. An increase in energy expenditure was observed in a small study of older men after moderate coffee consumption [44]. Furthermore, a study conducted by Engels and Haymes (1992) [45] showed increases in pre-exercise free fatty acids, glycerol, and lactate concentrations in sedentary men after a single dose of caffeine (5 mg·kg^−1^). However, if free fatty acids are the main source of fuel, lactate levels should not increase.

Finally, the physiological significance of the inverse association of LPE 16:0 with caffeinated and decaffeinated coffee consumption requires further work hypotheses and research to be determined.

Allantoin is produced from the nonenzymatic oxidation of uric acid in humans and is considered to be a specific biomarker of oxidative stress [46]. It has been hypothesized that the diuretic action of caffeine might lower blood uric acid concentrations [47] and subsequent allantoin production. However, in our study lower plasma levels of allantoin were associated with decaffeinated coffee drinking.

The results of the present study should be interpreted in the context of its limitations and strengths. First, although the FFQ used to assess coffee consumption was validated, misclassification bias cannot be completely excluded. Secondly, even though we used a comprehensive metabolic analysis to cover a wide range of metabolites, some of those previously associated with coffee consumption were neither selected by the model nor identified in the platforms we used. Thirdly, the cross-sectional design does not allow making any causal inference of the observed associations, and therefore both directions are plausible. Finally, participants were elderly Mediterranean individuals at high cardiovascular risk consuming low amounts of coffee per day and this may limit the generalizability of the findings to other age-groups or populations. Regarding strengths, we have used a multimetabolomics approach in order to analyze a wide range of biochemical compounds in a relatively high sample size and have internally cross-validated our results.

In conclusion, different sets of plasma metabolites were associated with the consumption of 50 mL or more per day of total, caffeinated and decaffeinated coffee in a Mediterranean population at high cardiovascular risk. These sets consisted of caffeine; 5-acetylamino-6-amino-3-methyluracil; cotinine; lipid species, such as sphingomyelin, phosphatidylethanolamine, and phosphatidylcholine; and other metabolites including alpha-glycerophosphate, hippurate, kynurenic acid, glycocholate, lactate, and allantoin. Plasma caffeine appeared to best discriminate total and caffeinated coffee consumers versus nonconsumers. Despite a low discriminative ability of decaffeinated coffee observed from the multimetabolite model this suggests that other potential metabolites could be used as candidate biomarkers of consumption. Therefore, our comprehensive metabolic analysis did not result in new, reliable potential set of metabolites for coffee consumption.At least in our population the selected metabolites did not accurately discriminate coffee consumers. Dietary questionnaires remain practical and one of the most affordable way to gather coffee intake data. Further studies are needed for assessing the involvement of the identified metabolites in health or disease.

## Figures and Tables

**Table 1:** Characteristics of the study subjects according to coffee and types of consumption.

	Non-Coffee Consumers	Total Coffee Consumers	Caffeinated Coffee Consumers	Decaffeinated Coffee Consumers	Total Subjects
Characteristic	*n* = 285	*n* = 1379	*n* = 512	*n* = 721	*n* = 1664
Coffee consumption (mL/day) *	0 (0, 0)	50 (50, 475)	50 (50, 250)	50 (50, 350)	50 (0, 475)
Male sex, N (%)	102 (35.8)	591 (42.9) ^b^	257 (50.2) ^b^	265 (36.8)	693 (41.6)
Age (years)	67.64 ± 6.25	67.04 ± 5.94	66.32 ± 5.93 ^a^	67.71 ± 5.94	67.14 ± 6
Body mass index (kg/m^2^)	29.42 ± 3.42	30.03 ± 3.62 ^a^	29.7 ± 3.52	30.25 ± 3.7 ^a^	29.92 ± 3.59
Waist circumference (cm)	99.61 ± 9.9	100.29 ± 10.22	100.33 ± 9.67	100.26 ± 10.56	100.17 ± 10.17
Smoking, N (%)					
Never	196 (68.8)	793 (57.5) ^b^	262 (51.2) ^b^	461 (63.9)	989 (59.4)
Former	58 (20.4)	344 (24.9)	131 (25.6)	173 (24.0)	402 (24.2)
Current	31 (10.9)	242 (17.5)	119 (23.2)	87 (12.1)	273 (16.4)
Type 2 diabetes, N (%)	80 (28.1)	375 (27.2)	150 (29.3)	189 (26.2)	455 (27.3)
Dyslipidemia, N (%)	202 (70.9)	1077 (78.1) ^b^	411 (80.3) ^b^	553 (76.7)	1279 (76.9)
Hypertension, N (%)	254 (89.1)	1192 (86.4)	426 (83.2) ^b^	640 (88.8)	1446 (86.9)
Family history of CVD, N (%)	79 (27.7)	338 (24.5)	123 (24.0)	188 (26.1)	417 (25.1)
Cardiac medication, N (%)	25 (9)	122 (9.1)	42 (8.4)	72 (10.3)	147 (9.1)
Antihypertensive agents, N (%)	211 (74.6)	1034 (75.1)	360 (70.5)	566 (78.7)	1245 (75)
Lipid-lowering medication, N (%)	120 (42.3)	653 (47.5)	225 (44)	358 (49.8)	773 (46.6)
Insulin medication, N (%)	8 (2.8)	57 (4.1)	16 (3.1)	32 (4.5)	65 (3.9)
Oral antidiabetics, N (%)	51 (18)	262 (19)	112 (21.9)	122 (17)	313 (18.9)
MedDiet score	8.73 ± 1.86	8.63 ± 1.86	8.54 ± 1.93	8.74 ± 1.83	8.65 ± 1.86

Data shows mean ± SD or number (%).* median (min, max). ^a^ *p*-value < 0.05 (Student’s *t*-test between coffee categories). ^b^ *p*-value < 0.05 (X^2^ between coffee categories).

**Table 2:** Metabolites ranked from the highest to the lowest elastic net positive regression coefficients for coffee and its types consumption.

Total Coffee	Caffeinated Coffee	Decaffeinated Coffee
AAMU 0.462	Caffeine 0.545	Hydroxyhippurate 0.065
Caffeine 0.330	AAMU 0.140	Alpha-glycerophosphate 0.047
Cotinine 0.022	C24:0 SM 0.031	C24:0 SM 0.018
C24:0 SM 0.015	Cotinine 0.015	Hippurate 0.014
		C40:6 PC 0.006

Metabolites selected at least once after running the elastic net model 10 times using the parameter “lamda.min” inthe case of total and decaffeinated coffee or “lambda.1se” in the case of caffeinated coffee after the “cv.glmnet” function procedure. Abbreviations: AAMU, 5-Acetylamino-6-amino-3-methyluracil; SM, sphingomyelin; PC, phosphatidylcholine. Total coffee: consumers ≥50 mL/day (*n* = 1379) vs. nonconsumers (*n* = 285); caffeinated coffee: consumers ≥50 mL/day (*n* = 512) vs. nonconsumers (*n* = 285); decaffeinated coffee: consumers ≥50 mL/day (*n* = 721) vs. nonconsumers (*n* = 285).

**Table 3:** Metabolites ranked from the highest to the lowest elastic net negative regression coefficients for coffee and its types consumption.

Total Coffee	Caffeinated Coffee	Decaffeinated Coffee
Proline betaine −0.031	Sucrose −0.062	C16:0 LPE −0.025
Kynurenic acid −0.018	Proline betaine −0.019	Phosphocreatine −0.017
Glycocholate −0.016	Acetaminophen −0.017	Allantoin −0.009
Lactate −0.016	C16:0 LPE −0.011	
Glyco-deoxy-chenodeox −0.013	Piperine −0.006	
Sucrose −0.007	Hypoxanthine −0.002	
7-methylguanine −0.006		

Metabolites selected at least once after running the elastic net model 10 times using the parameter “lamda.min” in the case of total and decaffeinated coffee or “lambda.1se” in the case of caffeinated coffee after the “cv.glmnet” function procedure. Abbreviations: LPE, lyso-phosphatidylethanolamine. Total coffee: consumers ≥50 mL/day (*n* = 1379) vs. nonconsumers (*n* = 285); caffeinated coffee: consumers ≥50 mL/day (*n* = 512) vs. nonconsumers (*n* = 285); decaffeinated coffee: consumers ≥50 mL/day (*n* = 721) vs. nonconsumers (*n* = 285).

## Abbreviations

AAMU	5-acetylamino-6-amino-3-methyluracil
alpha-GP	alpha-glycerophosphate
AUC	area under the curve
IFN-γ	interferon-γ
LC–MS	liquid chromatography tandem mass spectrometry
LC-CoA	long-chain coenzyme A
LPE	lyso-phosphatidylethanolamine
SM	sphingomyelin
TAG	triacylglycerol
